# The Role of Hypoxia and SRC Tyrosine Kinase in Glioblastoma Invasiveness and Radioresistance

**DOI:** 10.3390/cancers12102860

**Published:** 2020-10-04

**Authors:** Filippo Torrisi, Nunzio Vicario, Federica M. Spitale, Francesco P. Cammarata, Luigi Minafra, Lucia Salvatorelli, Giorgio Russo, Giacomo Cuttone, Samuel Valable, Rosario Gulino, Gaetano Magro, Rosalba Parenti

**Affiliations:** 1 Department of Biomedical and Biotechnological Sciences (BIOMETEC), Section of Physiology, University of Catania, 95123 Catania, Italy; filippo.torrisi@unict.it (F.T.); nunziovicario@unict.it (N.V.); federica.spitale94@gmail.com (F.M.S.); rosario.gulino@unict.it (R.G.); 2 Institute of Molecular Bioimaging and Physiology, National Research Council, IBFM-CNR, 90015 Cefalù, Italy; luigi.minafra@ibfm.cnr.it (L.M.); giorgio.russo@ibfm.cnr.it (G.R.); 3 Department G.F. Ingrassia, Azienda Ospedaliero-Universitaria “Policlinico-Vittorio Emanuele” Anatomic Pathology, University of Catania, 95125 Catania, Italy; lucia.salvatorelli@unict.it (L.S.); g.magro@unict.it (G.M.); 4 National Laboratory of South, National Institute for Nuclear Physics (LNS-INFN), 95125 Catania, Italy; cuttone@lns.infn.it; 5 ISTCT/CERVOxy Group, GIP Cyceron, CEA, CNRS, Normandie Université, UNICAEN, 14074 Caen, France; samuel.valable@cnrs.fr

**Keywords:** Glioblastoma, hypoxia, radioresistance, invasion, SRC tyrosine kinase, targeted therapy

## Abstract

**Simple Summary:**

The biological pathways underlying glioblastoma malignancy and radioresistance are still unclear. In this review, we describe the role of the hypoxic microenvironment and SRC proto-oncogene non-receptor tyrosine kinase in the activation of radioresistance and invasion pathways of glioblastoma. We also highlight the hypoxia- and ionizing radiation-induced infiltration, providing updated evidences on the involvement of SRC in these processes. Optimizing radiotherapy and identifying druggable molecular players are crucial steps to improve current glioblastoma therapeutic strategies.

**Abstract:**

Advances in functional imaging are supporting neurosurgery and radiotherapy for glioblastoma, which still remains the most aggressive brain tumor with poor prognosis. The typical infiltration pattern of glioblastoma, which impedes a complete surgical resection, is coupled with a high rate of invasiveness and radioresistance, thus further limiting efficient therapy, leading to inevitable and fatal recurrences. Hypoxia is of crucial importance in gliomagenesis and, besides reducing radiotherapy efficacy, also induces cellular and molecular mediators that foster proliferation and invasion. In this review, we aimed at analyzing the biological mechanism of glioblastoma invasiveness and radioresistance in hypoxic niches of glioblastoma. We also discussed the link between hypoxia and radiation-induced radioresistance with activation of SRC proto-oncogene non-receptor tyrosine kinase, prospecting potential strategies to overcome the current limitation in glioblastoma treatment.

## 1. Introduction

**Figure 1:**
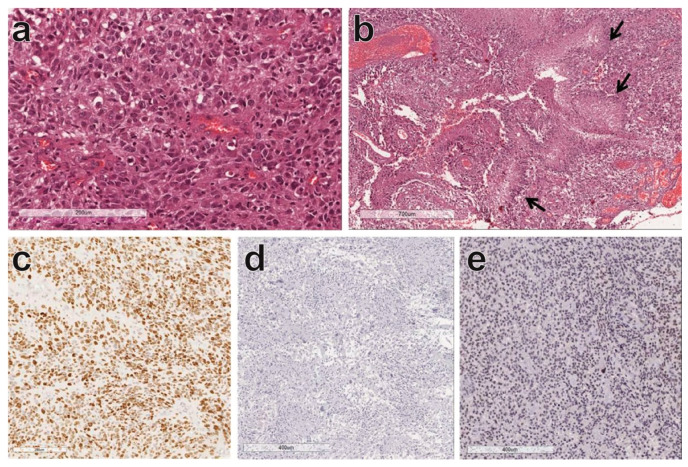
Glioblastoma, isocitrate dehydrogenase (IDH) wildtype. Highly anaplastic glial cells with nuclear atypia and pleomorphism (**a**); palisading necrosis (arrows) and microvascular proliferation (**b**); at immunohistochemistry the neoplastic cells show a high proliferation index (Ki67); (**c**) no immunostaining for IDH-1; (**d**) and retained ATRX chromatin remodeler (ATRX) (**e**).

Glioblastoma (GBM) is the most frequent and aggressive primary brain tumor with an incidence of 5/100,000 per year and a median survival of 12−15 months after diagnosis, despite aggressive multimodal treatments [1]. Recent genetic and molecular advances on GBM cellular states provided both genetic and micro-environmental determinants, establishing four GBM subtypes recapitulating astrocyte-like, mesenchymal-like, neural-progenitor-like, and oligodendrocyte-progenitor-like phenotypes [2,3]. Such a classification specifies molecular and genetic profiles associated with GBM subtypes, thus providing additional information to the histopathological characterization in accordance with World Health Organization guidelines [4]. Histologically, GBM is a highly cellular glioma composed by glial cells with marked nuclear atypia and pleomorphism (Figure 1a). Common typical diagnostic features are microvascular proliferation (Figure 1b), often with glomerular-like appearance and palisading necrosis characterized by regular areas of necrosis surrounded by dense accumulations of neoplastic cells (Figure 1b). Proliferative activity is usually prominent with highly mitotic count. The proliferation index is evaluated immunohistochemically by analyzing the proportion of cells expressing the nuclear markers of proliferation Ki-67, accounting for a total of 15−20% of GBM cells, even if some tumors show a proliferation index greater than 50% (Figure 1c). Two different molecular types of GBM are recognized: GBM isocitrate dehydrogenase (IDH)-wildtype and GBM IDH-mutant, which are commonly associated with primary and secondary GBM, respectively. Indeed, based on mutation of other genes, in GBM IDH-wildtype, the gliomagenesis occurs early due to the amplification/mutation of epidermal growth factor receptor (EGFR) and the loss of the phosphatase and tensin homolog (PTEN) gene. In GBM IDH-mutant, the mutation of tumor protein p53 (TP53) and the deletion of 1p/19q determine the acquisition of the genetic alteration, resulting in a lower grade astrocytoma or oligodendroglioma.

GBM IDH-wildtype is more frequent, usually occurs in older patients (mean age: 62 years), and it is characterized by absence of mutated IDH-1 (Figure 1d) and expression of ATRX chromatin remodeler (ATRX, Figure 1e) is expressed. Conversely, GBM IDH-mutant, is less frequent and develops in significantly younger patients (mean age 45 years). It may arise from a lower grade glioma (diffuse or anaplastic astrocytoma) and shows IDH-1 mutation and loss of ATRX.

Many advances have been made to elucidate the biological mechanisms promoting GBM development and progression, including genetic mutations, metabolism, and the microenvironment role. A common denominator is the hypoxic microenvironment that characterizes this scenario, feeding the renewed players of the tumor set. Therefore, hypoxia and associated necrosis have provided this tremendous neoplasm with an identity card, showing salient marks of the different subtypes and stages of invasiveness and aggressiveness.

Despite recent evidence expanding the current knowledge on GBM, therapeutic options for newly diagnosed cases are still limited to surgery, standard chemotherapy (i.e., temozolomide), and radiotherapy [1]. Indeed, clinical reports showed that radiotherapy combined with temozolomide improves the overall survival of patients, after surgical resection [5]. Current guidelines indicate radiotherapy dosing up to 60 Gy for 30 fractions (2 Gy/day) as the best approach to reduce radiotherapy-induced side effects and to counteract radioresistance and recurrences [6]. Hypofractionated treatment of 40 Gy in 15 fractions over 3 weeks is suggested only for patients older than 70 years old and with poor performance status [7]. 

However, in this context, in order to reduce GBM aggressiveness and to simultaneously increase the effect of the radiation dose, there is an urgent clinical need to develop targeted therapy and radiosensitizing agents. Strategies to reach this aim should take into account two main features of GBM: hypoxia and invasiveness. These two features are also correlated with each other; indeed, hypoxia is known to support GBM radioresistance and it is also involved in increased GBM invasiveness and infiltration into the surrounding tissue [8].

In this sense, it is essential to dissect hypoxia-related events which play a central role in determining cancer cell invasiveness and infiltration into the surrounding tissue, and also in causing radioresistance. The investigation of molecular mechanisms may elucidate the relationship between GBM hallmarks and hypoxia, providing new key molecular targets.

In this review we describe the role of hypoxia and the molecular mechanisms involved in GBM invasiveness and radioresistance, focusing on the involvement of SRC proto-oncogene non-receptor tyrosine kinase (SRC). We also report potential strategies to improve efficacy of radiotherapy against hypoxia, invasiveness, and SRC activation. 

## 2. SRC Proto-Oncogene Non-Receptor Tyrosine Kinase and Glioblastoma

**Figure 2:**
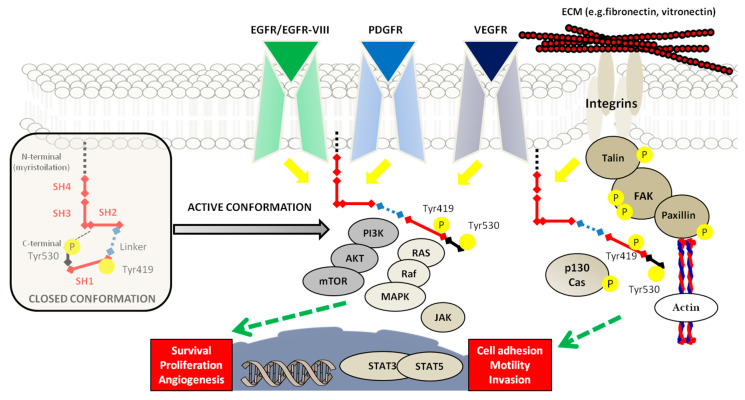
Schematic representation of SRC structure and regulation. The inactive form of SRC is illustrated on the left side, with the specification of each SH domain; in this closed conformation, the phosphorylation of Tyr530 on C-terminal creates a link with the SH2 and the catalytic site, which is positioned on SH1, becoming not accessible for the substrates. In the transition to the active form, the phosphorylation of Tyr419 is showed with the main pathways that act by downstream and upstream effectors. The conformational switch is mediated by many phosphatases, such as PTPα, PTPγ, SHP-1 and -2, and PTP1B, able to dephosphorylate SRC. The regulation of activated SRC is displayed with the RTKs and integrins signaling. In particular, the downstream effectors of RTKs/SRC interaction lead to target genes transcription for survival, proliferation, and angiogenesis sustainment. The interaction of integrins with ECM components and their localization on cell adhesion sites, determines the modulation of cell motility: The SRC signaling pathway induces a cascade that results in the phosphorylation of several proteins, such as FAK, talin, and paxillin, with the final actin cytoskeleton regulation that is responsible for migration and invasion mechanisms.

Previous studies revealed that SRC is shaping GBM pathophysiology and features such as proliferation, migration, invasiveness, and angiogenesis [9]. SRC is composed of 4 SRC homology domains (SH): SH4 is linked to N-terminal with a 14-carbon myristic acid moiety, a unique domain different for all members and whose function is far to be fully elucidated, SH3 is a non-catalytic domain and SH2 linked, with a SH2-kinase linker, to the SH1 domain, containing a kinase domain involved in the activation of SRC autophosphorylation at the level of the tyrosine residue (Tyr419), followed by a C-terminal negative regulatory domain (Tyr530) [10]. In particular, the autophosphorylation of Tyr419 switches the protein from an inactive to an active conformation, whereas the phosphorylation of Tyr530 determines the binds of the SH2 domain and the inhibition of protein kinase activity. There are various hypotheses to explain the aberrant activation mechanisms of SRC in tumors that mostly concern the destabilization of the SH4-SH3-SH2-Linker-SH1, leading to the promotion of adhesion, invasion, and motility. Indeed, SRC protein can be activated by the direct binding of the SH2 and SH3 domains with other surface receptors, such as integrins, with cytoplasmatic tyrosine kinases, such as focal adhesion kinase (FAK), or with the cytoplasmic portion of activated receptor tyrosine kinases (RTKs), which hinder the inhibitory SRC interactions [11]. The integrin/FAK/SRC axis regulates intercellular interaction and communication between cells and the extracellular matrix (ECM) in a signal transduction manner. Integrins and FAK colocalize on the focal adhesions, and SH2 and SH3 domains are respectively high affinity sites for binding with the autophosphorylation domain and with proline-rich regions of the FAK. On one side, the interaction of FAK with the SH2 domain of SRC displaces the salt bridge formed after Tyr530 phosphorylation in the closed conformation and leads to activation of SRC. Conversely, following the SRC-FAK bond, SRC phosphorylates two tyrosine residues on the FAK kinase domain, increasing their kinase activity. The FAK-SRC complex phosphorylates the serine and threonine sites of paxilline, which regulates the Rho family of GTPases, such as RhoA, promoting actin-stress-fiber formation in order to regulate the structural organization of the cytoskeleton for adhesion, motility, and cell division [12]. In addition, SRC phosphorylates tyrosine residues of the C-terminal of FAK which acts as a binding site for other molecules that regulate communication signaling between cells or between cells and ECM [13]. In particular, these processes are mediated by the formation of the FAK-SRC complex that regulates guanine-exchange factors and GTPase-activating proteins, leading to membrane protrusion or cytoplasmatic projections formation such as filopodia [14]. Furthermore, the activation of SRC mediated by RTKs, through the interaction with SH2 domains or the recruitment of small GTPases Ras/Ral and the inhibition of the Csk negative regulator, leads to downstream multiple effectors, such as PI3K/Akt, Ras/Raf/MAPK, STAT3/STAT5B, and p130 Cas pathways, which are respectively involved in survival, proliferation, angiogenesis, and motility [15] (Figure 2). 

Since the discovery of SRC as a proto-oncogene, the role of SRC in cancers has been largely investigated, and due to the rare cases of gene mutation and amplification, it has remained unclear for a long time. Then, much evidence supported the oncogenic role of SRC mainly due to the interaction with various signaling molecules activating pathways for the promotion, maintenance, and progression of several cancers. Deregulation of SRC was not only associated with central nervous system cancers, but also with several others, including prostate, colorectal, breast, lung, head-neck, and pancreatic cancers [16]. In addition to SRC, also other proteins among the non-receptor tyrosine kinase family have been associated with tumor development, including Fgr, Fyn, Yes, and Lyn [16].

In GBM, the absence of gene amplification and mutation confirmed that the hyperactivation of SRC is linked to aberrant activation of RTKs and surface receptors [17]. Indeed, FAK and other RTKs, including epidermal growth factor receptor (EGFR), platelet-derived growth factor receptor (PDGFR), and vascular endothelial growth factor receptor (VEGFR), determine the loss of SRC interdomains interactions involved in SRC inhibition, leading to most of the GBM-associated phenomena [9,18,19,20]. The role of SRC in GBM progression is not only directly linked to the main proliferation and survival pathways affected by deregulation of downstream RTKs; indeed, it was also found that SRC modulates the activation or the overexpression of proinflammatory transcription factors, contributing to an increase in aggressiveness and support of the complex tumor microenvironment [21]. The microenvironment has a key role in GBM; cancer cells establish a complex network with reactive stroma composed by a heterogenic cell population, including immune cells, fibroblasts, precursor cells, endothelial cells, macrophages, lymphocytes, as well as signaling molecules and ECM components [22]. For these reasons, SRC signaling in GBM holds great promise and may provide crucial insight into developing new therapeutic approaches. 

## 3. Hypoxia and Glioblastoma

Despite hypoxia being usually associated with cell suffering and death, it has a different connotation in solid tumors, representing a common feature of increased malignancy. In fact, hypoxia can trigger the production of inflammatory mediators which potentiate neoplastic risk [23]; furthermore, in response to hypoxia, tumor tissues activate the production of VEGF, which is one of the main downstream targets of the HIF-1α pathway, increasing vascular permeability and promoting angiogenesis. The creation of new vessels is fundamental for the stromal blood supply in order to maintain the rate of cell growth [24].

Intratumoral oxygen pressure (pO_2_) values in GBM represent a critical aspect of the radiotherapy approach. The aerobic value of the brain tissue is of about 40 mmHg in physiological conditions, whereas it has been shown to be significantly lower in GBM [25]. To be defined hypoxic, a tissue must reach a pO_2_ value below 10 mmHg, which is the result of the unbalanced oxygen supply and consumption rate [26]. In GBM, hypoxia ranges from mild (pO_2_ = 20 to 4 mmHg) to severe condition (pO_2_ = 4 to 0.75 mmHg), especially in necrotic and micronecrotic areas [26]. Hypoxia occurs when the distance to the nearest blood vessel is impeding appropriate exchanges but also when blood perfusion is altered. In general, both phenomena occur in GBM and it is considered that chronic but also cycling hypoxia take place, making it very difficult to deal with such a complex scenario [27]. 

## 4. Hypoxic Regulation of SRC in Glioblastoma Development and Invasion

Hypoxia seems to play a major role in the SRC tyrosine-kinase pathway, which is constitutively activated in several malignant human tumors, including GBM [28,29,30]. In fact, all the RTKs described above are targets of the transcription factors hypoxia-inducible factor-1α (HIF-1α), which is induced under conditions of low oxygen. The oxygen-sensitive subunits of HIF transcription factors are normally synthesized in normoxic condition, but they are unstable and targeted for ubiquitination and degradation by the von Hippel–Lindau protein (VHL). VHL is able to recognize HIF-1α/HIF-2α thanks to their hydroxylation that is performed by prolyl hydroxylases, which use molecular oxygen as a cofactor; for this reason, under hypoxic condition, HIF-1α and HIF-2α cannot be hydroxylated and they bind the HIF-1β subunit, allowing gene transcription regulation [31]. Indeed, as early as 1995, it has been shown that phosphorylated SRC protein is highly active in GBM cells, particularly under hypoxic conditions [32]. In this study, it has also been shown that the increase in SRC activity in hypoxia causes the VEGF upregulation, which therefore represents a downstream transcription of the SRC pathway induced by hypoxia [32]. Moreover, a correlation between angiogenesis and hypoxia was also sustained by the observation of a significant increase in vascularization related to the hypoxia-signaling pathway involving integrin upregulation [33,34]. The integrin overexpression in hypoxic GBM cells was correlated to the activation of FAK, which promotes the activation of small GTPase such as RhoB. RhoB increases the phosphorylation leading to the inhibition of glycogen synthase kinase-3β (GSK-3beta) pathway, involved in the degradation of HIF-1α [35]. This evidence supported angiogenesis inhibition as a strategy for GBM therapy; however, it was shown that in response to the anti-VEGF antibody (Bevacizumab), further cell survival mechanisms were activated due to increased SRC signaling [36]. The robust invasion in response to anti-VEGF may be, at least partially, associated with neo-vascular loss, low perfusion, and consequent hypoxia, which induces SRC activation [37]. In addition to angiogenesis, metabolism alteration has been identified as a typical hallmark of GBM, mainly due to the hypoxic condition that promotes the upregulation of glycolysis by HIFs and sustains the so-called Warburg effect [38]. In this scenario, there is not a direct link between the metabolism alteration and the SRC activity in GBM; among the factors influencing GBM metabolism, the MYC oncoprotein has been shown to increase glycolysis in GBM and its regulation has been associated with the SRC pathway in other tumors. Therefore, there is likely an involvement of the SRC-MYC axis in driving metabolic reprogramming, in addition to the RTKs expression by HIF-1α [39].

GBM is a highly infiltrating tumor characterized by intense proliferation, the ability to invade surrounding tissue, and dysregulated biological pathways operating in both intra- and extra-cellular compartments. Among the most crucial alterations, the dysfunction of cellular metabolism leads to a series of consecutive events which invariably affect the degree of malignancy. In particular, hypoxic conditions are known to control the expression of target genes such as VEGF, TGF-β2, MMP-1,2, and 9, human plasminogen activator inhibitor type 1, endothelin-1, and erythropoietin (EPO), influencing angiogenesis, tumor growth, and GBM invasiveness [40,41,42]. 

Hypoxia also supports a complex remodeling of cytoskeleton, which includes a number of linked events such as (i) alteration of cell adhesion, (ii) activation of cell motility, (iii) production of proteolytic enzymes. Cell adhesion modification occurs through the modulation of E-cadherin expression, which is commonly altered in tumors [43], generally as a result of mutation or gene suppression by hypermethylation [44]. It has been reported that E-cadherin expression decreases in high grade brain tumor as compared to healthy tissue [45]. In particular, a shift occurs from E-cadherin to N-cadherin expression, which increases the interaction between cancer and stromal cells [46], promoting the activation of cell motility as part of the complex epithelial-mesenchymal transition (EMT) [46]. Several pathways are involved in the cadherin switching, consisting in the upregulation of N-cadherin, which creates less efficient adherent junctions than E-cadherin. In this context, it has been demonstrated that zinc finger E-box binding homeobox 1 (ZEB1) was upregulated in U87 cells under hypoxic conditions, with the consequent nuclear accumulation with HIF-1α and HIF-2α. Roundabout guidance receptor 1 (ROBO) is a downstream effector of ZEB1, which takes part in the process of loss of N-cadherin adhesion to the cytoskeleton, thus promoting motility and finally supporting the EMT process [47,48].

After cell adhesion loss, cancer cells increase their motility by a number of processes such as stimulating the activity of cytoskeleton, autocrine/paracrine chemotaxis or proteolysis activity, and ECM degradation [49]. Cancer cells are stimulated to move via interactions between adhesion molecules (i.e., integrins) and the products of ECM degradation. Under hypoxic condition, GBM cells increase interactions between the mutated form of epidermal growth factor receptor vIII (EGFR-vIII) and αvβ3 and αvβ5 integrins [50,51] which are recruited on the cell membrane surface, leading to invasion enhancement mediated by FAK activation [35]. Such a process generates the so-called adhesion plate, where integrins interact with FAK promoting cytoskeleton contraction and proliferative effects by intracellular signal transduction. It is noteworthy that phosphorylation of FAK is induced in hypoxia by a pathway that involved the procollagen-lysine 2-oxoglutarate 5-dioxygenase (PLOD2) [52].

The production of proteolytic enzymes is a crucial event during invasion. In particular, increased activity of matrix metalloproteases (MMPs) is associated with higher grade glioma and correlated with shorter overall survival in GBM patients [53,54], even if in vitro studies on GBM cell lines provided evidence of a heterogeneous expression of MMPs [55,56]. On this aspect, a well-characterized effect is mediated by hypoxia. Indeed, low oxygenation indirectly promotes MMP-9 and MMP-2 upregulation and increased proteolytic activity, by reducing pH levels in the tumor microenvironment. This condition is related to the increased metabolic activity of the tumor that, based on glycolysis in hypoxic conditions, increases the lactic acid levels by gradually reducing the pH [57]. In addition, induction of type A lactate dehydrogenase (LDH-A), regulating the transforming growth factor-β2 (TGF-β2), has been shown to trigger the cascade of transcriptional regulation of MMP-2 and integrin αvβ3 expression, strongly influencing the tumor invasiveness [58]. It is noteworthy that the tissue inhibitor of metalloproteases (TIMP) and TIMP-like molecules, which are synthesized and released by resident cells, counteracting ECM degradation including MMPs, inhibit GBM invasion [59,60].

**Figure 3:**
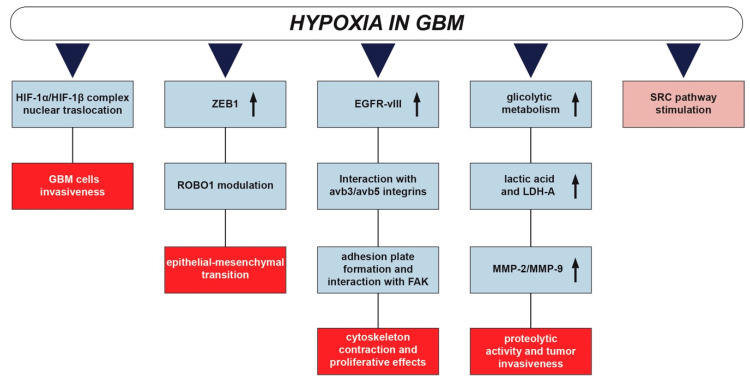
Schematic representation of the main pathways for the invasion process induced by hypoxia. SRC pathway stimulation under hypoxia contributes to the deregulation of the principal events required for invasion, including cell adhesion, activation of cell motility, and production of proteolytic enzymes.

SRC drives GBM invasion and progression [9,61]. The hypoxia-induced SRC pathway entirely influences the process described above, finally resulting in fostered invasiveness. In fact, it primarily involves EGFR-vIII and integrin β3 interaction, the recruitment of αvβ3 integrin on GBM cell membranes and the creation of focal adhesion complexes by FAK activation [62]. Finally, the EGFRvIII / integrin β3 / FAK / SRC axis leads to the activation of the intracellular signaling pathway ERK1/2, MAPK, AKT, and STAT3, which determines the upregulation of MMP-2 and MMP-9, further promoting cell invasion [63]. It is also interesting that the SRC-induced TGFβ pathway activation via α-SMA is associated with the promotion of cancer-associated fibroblasts (CAFs), which further increase chemotactic mediated migration of GBM cells (Figure 3) [30,64].

## 5. Hypoxia-SRC Axis Promoting Glioblastoma Radioresistance

Hypoxia-induced radioresistance in GBM is a radiobiological event due to the interaction between ionizing radiation (IR) and the biological matter. IR can determine direct and indirect damage to all organelles and macromolecules of cells [65]. IR induces single strand breaks, or double strand breaks, directly on DNA molecules, which are difficult to repair and are associated with oxygen-independent-cell death. Vice versa, indirect damage is closely linked to the presence of oxygen. Indeed, IR interacting with water molecules induces the formation of reactive oxygen species (ROS) through a radiolysis reaction, which is much more efficient in well oxygenated tissues that facilitate the formation of superoxide radical and hyperoxide, leading to the amplification of damage and increased radiotherapy efficiency [66]. In particular, according to oxygen fixation hypothesis, increasing ROS concentration induces the so-called “fixed damage from oxygen” on DNA, invariably leading to cell death [67]. In hypoxic areas, the effect of cell death induced by ROS and oxygen reactions is less efficient, with the resulting radioresistance. In view of the crucial significance of the GBM hypoxic condition, the “oxygen effect” and the response to radiotherapy treatment is assessed by the oxygen enhancement ratio (OER) parameter, which is defined as the ratio between the dose in hypoxia and normoxia to reach the same biological effect [68].

It has been shown that the majority of GBM recurrences occur at the margins of surgical resection or within the high dose irradiation field, likely associated to residual cells that receive a sublethal irradiation and escape from the primary tumor, while underlying molecular mechanisms remained partially uncovered [69,70]. Moreover, the high incidences of recurrences within the high-dose irradiation field, in close proximity (1–2 cm) to the primary tumor, is associated to the existence of a subpopulation of resistant cells with stem cell-like properties, called glioblastoma stem cells (GSCs), which are promoted in the high hypoxic site or niches [71,72]. It was reported that IR promoted the phenotypical switch from neural to mesenchymal types in GSCs in recurrences; the IR induces the production of proinflammatory factors or NF-κB and induction of C/EBP-β, which in turn activates CD109 transcription binding its promoter. CD109 is a clear marker of the mesenchymal subtype [73]. GSCs were also implicated in the formation of new blood vessels in response to IR, enhancing their trans-differentiation in tumor derived endothelial cells, by the activation of the Tie2 signaling pathway [74]. SRC was found highly expressed in GSCs, where they can enhance the migratory ability [75] and potentiate the stemness properties being a downstream target, together with transcription 3 (STAT3)-Kirsten rat sarcoma viral oncogene homolog (KRAS), in the MerTK pathway. Indeed, MerTK is upregulated in GBM and it was reported that the silencing of KRAS and SRC suppressed mesenchymal markers and GSC features in MerTK-overexpressing X01 GBM stem-like cells [76]. 

Besides being active during hypoxia, SRC activation has been found to promote invasiveness and motility of cancer cells in response to radiotherapy; in breast cancer cells it has been shown that fractional irradiation caused an increase in SRC phosphorylation [77]. In the same study, it has been observed that SRC inhibition reduced cell migration and the expression of markers associated with the EMT process [77]. The activation of malignant phenotypes of GBM in response to radiation was reported through the induction of MMP-2, involving pathways mediated by the interaction of SRC with EGFR. In this study, it has been reported that IR induced phosphorylation of SRC kinase and that SRC inhibition by PP2 reduced MMP-2 secretion, AKT activation, and SRC phosphorylation in irradiated cells. Moreover, PP2 was able to block IR-induced EGFR phosphorylation, whereas inhibition of EGFR did not affect the phosphorylation of SRC, identifying the possibility that radiation may stimulate the SRC activation regardless of EGFR/AKT pathway [78]. It has been also reported that IR-induced invasion modulating the ECM protein, is not only due to MMP action, but also to high production of other components such as hyaluronic acid, which acts as an extracellular signaling molecule for the mesenchymal shift of GBM, in response to radiation; hyaluronic acid is recognized by the CD44 receptor, which is a clear marker of the mesenchymal subtype. The interaction of hyaluronic acid and the CD44 receptor, leads to SRC activation, promoting tumor progression and radioresistance [79]. Moreover, IR-SRC activation promotes invasion processes also due to FAK, ephrin type-A receptor 2 (EphA2), and EGFR-vIII signaling [80]. The EGFR-vIII expressing cells have been shown to release ligands such as hepatocyte growth factor (HGF) and interleukin 6 (IL6), activating SRC in EGFR expressing cells, thus increasing diffusion and infiltration [81]. 

The SRC pathways induced by IR have been also evaluated in relation to the intercellular communication systems in the context of signal molecules transmission by connexin-based channel and extracellular vesicles [82,83,84]. It has been shown in vitro that connexin43 (Cx43)-gap junction and -hemichannel activity is implicated in invadopodia formation and function responsible for invasion capacity and MMP-2 activity by Cx43 dynamic interactions with partners including SRC [85,86]. It has also been shown that following irradiation, GBM cells can release exosomes, which stimulate the migration of recipient cells. In this condition, cells increase the expression of proteins involved in cell migration, including SRC, in addition to focal adhesion kinase (FAK), paxillin, and T neurotrophic tyrosine kinase receptor type 1 (TrkA) [87]. 

## 6. New Frontiers to Improve Radiotherapy: Evaluating the Potential of Synergistic Approaches

It is well known that hypoxia is associated with increased resistance to IR, contributing to treatment failures after radiotherapy based on X-rays. The need for new strategies to improve radiotherapy has become increasingly urgent and research efforts are currently focusing on studying synergistic approaches to overcome current limitations.

An action plan adopted to counteract hypoxia-induced radioresistance involves a model known as “hypoxia dose painting”, based on providing a personalized radiation dose according to local phenotypic or microenvironmental variations of the tumor, influenced by spatial and temporal heterogeneity of hypoxia [26,88]. Other aspects take into consideration the IR physical features including specific linear energy transfer (LET), which also have an impact on radiotherapy efficacy and biological effects. LET is a measure of ionization density and it is defined as the average energy (keV) transferred by a particle along the 1 μm path [89]. High LET particles show high ionization density, thus inducing increased direct cell damage, but display lower indirect effects mediated by ROS and other radicals [89,90]. Another main advantage of particle-based radiation therapy is the finite dose deposition in the tissue that allows sparing the normal brain tissue. Consequently, a frontier in radiotherapy is to combine multiple ion beams simultaneously, in order to deliver low-LET radiation in normoxic tumor areas and high-LET radiation in the hypoxic tumor microenvironment, in so doing optimizing IR-induced cell damage in a microenvironment-dependent manner [91]. Reoxygenation strategies have been also developed to improve radiotherapy efficacy both during the course of irradiation and by radiosensitizing drugs or nanoparticles delivered into the tumor to improve oxygenation [92,93]. 

Targeting the molecular mechanisms regulated by hypoxia represents a promising way to sensitize GBM cells to treatments. In general, the rationale to use radiosensitizing agents is to reduce the dose of IR maintaining similar biological effects in terms of cell death and reducing radiotherapy side-effects. Such a concept is expressed as dose modifying factor or sensitized enhancement ratio, both indicating the ratio between the dose alone and in the presence of the radiosensitizer to determine the same biological effect [94]. Radiosensitive agents also hold great potential to increase effectiveness of radiotherapy reducing OER with multivariate effects, such as blocking specific pathway induced by hypoxia, or enhancing DNA damage by affecting self-repairing mechanisms [95]. In addition to radiosensitive agents designed for specific biological targets, further promising candidates for synergistic approaches include sodium borocaptate (BSH) and boron phenylalanine (BPA). The combination of BSH/BPA with IR can determine an increase in therapeutic efficacy by increasing the LET, due to a selective accumulation of the Boron isotope ^10^B inside cancer cells that react with the thermal neutron to produce high-energy alpha particles, leading to the so-called boron neutron capture therapy (BNCT) [96]. Good results have been obtained, especially in Japan, thanks to imaging techniques labeling the BPA [97]; the main challenge for this promising therapy is not only related to the cost and availability of the neutron sources in clinical settings but also to the research of new boron carriers capable to cross the blood brain barrier [98]. A similar strategy using BSH/BPA combined with protons for proton boron capture therapy (PBCT) has revealed the possibility to enhance the proton therapy effectiveness, but preliminary results have been obtained and no clinical trials for GBM have been proposed so far [99].

Hypoxia induces a number of intracellular reactions such as the activation of the transcription factor HIF, which in turn activate a variety of cellular process in response to the lowering oxygen level [100]. Several molecular targets have been described as radiosensitizing agents in hypoxic conditions. For instance, EPO transcription is regulated by the HIF-1α/HIF-1β complex and it has a key role in GBM proliferation and survival through the AKT/PI3K pathway and the upregulation of Bcl-2/Bcl-xL anti apoptotic factors. Therefore, EPO receptor silencing not only increases the sensitivity of glioma cells to chemotherapy (temozolomide) as well as X-rays, but also counteracts the hypoxia-induced chemo- and radio-resistance [101]; for this reason, targeted therapy, such as specific antibodies, may be applied directly to EPO, EPO receptor, or to another downstream mediator of EPO receptor signaling pathway such as STAT3. Likewise, the hypoxic cell radiosensitizer doranidazole (PR-350) administration in malignant significantly enhanced radiation-induced reproductive cell death in vitro under hypoxia, suggesting a potent strategy for improving the clinical outcome of radiotherapy, reducing related side effects [102]. A promising strategy to enhance the radiosensitivity of GBM is represented by the application of targeted molecules that weaken the DNA damage response (DDR) signaling pathway. DDR can be considered as a group of highly interconnected signaling pathways, that cooperate to preserve the survival in response to the DNA damage by irradiation; DDR activation contributes to enhance radioresistance of GBM, which is able to reach high levels of double strand DNA break repair proficiency. The most representative agents belonging to this radiosensitizers group are inhibitors of the poly(ADP-ribose) polymerase (PARP) proteins; PARP are involved in DNA repair pathways, especially for DNA single-strand breaks [103]. Veliparib and olaparib are PARP inhibitors, largely evaluated at both the preclinical and clinical stages. However, despite some promising results, veliparib has not been shown to be effective in combination with temozolomide and radiotherapy in new diagnosed GBM [104]; clinical trials for olaparib are currently ongoing, and additional upstream or downstream DDR biomarkers, including DNA-dependent protein kinase and cell cycle checkpoint inhibitors are attractive target for the radiosensitization of GBM [105].

Beside radiosensitizing agents, novel strategies have also been tested as molecularly targeted drugs. Cilengitide is a drug that selectively blocks activation of the αvβ3 and αvβ5 integrins, amplifying the effect of IR and triggering an enhanced apoptotic response and tumor growth suppression [106]. Unfortunately, the results of two large phase-III clinical trials showed that combination of cilengitide, radiotherapy, and temozolomide for newly diagnosed GBM does not improve progression free survival and overall survival as compared to radiotherapy and temozolomide alone [107]. As previously reported, FAK participates with SRC in adhesion and migration signaling network; moreover, they are upregulated and activated in GBM influencing growth and motility. The combination of radiotherapy and FAK inhibition also provided promising results, showing radiosensitization in GBM cell lines in vitro [108]. Further studies encouraged the development of a potent, ATP-competitive, reversible inhibitor of FAK, called GSK2256098. A phase I clinical trial evaluated the tolerability for GBM treatment and additional clinical trials are evaluating the therapeutic efficiency of such an approach [109]. Likewise, inhibition of MMP-14 in combination with radiotherapy and temozolomide improved the survival of glioma-bearing mice as compared to single treatment group [110]; nevertheless, the main MMP inhibitor, marimastat, was tested with temozolomide, but not with radiotherapy, in a phase II trial for recurrent GBM [111]. 

SRC activation leads to different pathways activation, promoting cell adhesion, motility, survival, proliferation, and angiogenesis. Moreover, SRC is also activated in response to IR, promoting invasiveness and malignancy of GBM as a consequence. For this reason, SRC inhibition combined with RT represents a promising approach to increase the therapeutic effect as well as to block GBM progression. The SRC pathway is targeted by radiosensitizing strategies tested to treat GBM in preclinical studies or at different phases of active clinical trials. Several SRC inhibitors were tested to treat GBM and they have been recently reviewed by Cirotti et al. [21]. Noteworthy, dasatinib (Sprycel, by Bristol-Myers Squibb) was the most used in clinical trial. It is a dual inhibitor SRC/ABL proto-oncogene 1-non-receptor tyrosine kinase, also inhibiting other SRC family kinases, such as LYN proto-oncogene and FYN proto-oncogene SRC. In a single-arm phase II trial, dasatinib was tested as monotherapy and was considered ineffective to proceed to stage 2 [112]. The evidences of SRC inhibition to reduce invasiveness induced by anti-VGFA led to perform an additional trial, in which dasatinib was tested in combination with bevacizumab [36]. Even in this trial, dasatinib does not show a significant improvement as compared with bevacizumab alone [36]; no additional improvements were provided in combination with EGFR (erlotinib) [113] and cyclonexyl-chloroethyl-nitrosourea (CCNU) [114]. Recently, a clinical trial evaluating dasatinib in combination with temozolomide and radiotherapy on newly diagnosed glioblastoma did not show promising results (NCT00869401). The current efforts in evaluating SRC inhibition potential are coupled with research in drug design to develop optimized SRC inhibitors for combinatorial approaches with radiotherapy. Such a field benefits from the current knowledge on the limitations of previously tested drugs. For example, it is now clear that pharmacodynamic issues, such as overexpression of efflux transporters P-gp at the blood–brain barrier levels, strongly affects dasatinib efficiency [115]. Current efforts aim at the design of new SRC inhibitor drugs aiming at the optimization of combinatorial approaches with radiotherapy.

A new SRC inhibitor, belonging to the pyrazolo[3¨ -d] pyrimidines series (i.e., Si306, Lead Discovery Siena, Italy) showed an excellent pharmacodynamic profile and was able to significantly inhibit GBM cell growth in highly P-gp expressing cells as compared to dasatinib [116]. We previously demonstrated that Si306 showed a synergic radiosensitive effect with proton irradiation in GBM cell lines [117]. We also identified up- or down-regulated genes associated with the SRC pathway modulation in GBM cells after irradiation with proton therapy [117]. After 2 or 10 Gy irradiation with protons, we detected that the GBM cell cycle, motility, survival, and proliferation rate were strongly affected by Si306, also showing increased overall radiation efficiency [117]. Moreover, Si306 has been tested in combination with X-ray both in normoxic and hypoxic conditions, demonstrating a significantly increased effect as compared to radiotherapy alone [30,118]. These findings are encouraging the investigations on SRC mechanisms in order to discover a valuable approach to develop new effective therapy against GBM. Most of the trials with targeted therapy were conducted in patients with recurrent GBM and rarely were tested in combination with radiotherapy. Further studies and evidence from in vitro and preclinical studies could enhance the importance of molecularly targeted drugs in association with radiotherapy, increasing the number of clinical trials, in order to propose new solution to GBM treatment. 

## 7. Conclusions

The dynamic GBM profile is still limiting our knowledge on its progression and invasion. Nevertheless, the remarkable progress that is gradually being made allows us to have some clear conditions on which to focus our attention. Indeed, it is now widely accepted that the microenvironment, which can be defined hostile for its hypoxic and necrotic characteristics, paradoxically proves to be a survival stimulus for cancer cells able to reprogram molecules and pathways and above all migrate to new sites, so arguing, in short, the aggressive phenotype and invasiveness of the tumor.

Classical therapeutic approaches are facing strong limitations due to the intrinsic characteristics of GBM, such as heterogeneity, high invasiveness, and marked angiogenesis, but also due to physiological barriers protecting the central nervous system, such as the blood-brain barrier, and off target and side effects. The ideal approach therefore would be a synergistic combination of therapies specifically developed to counteract this aggressive brain tumor. Hypoxia-induced pathways dysregulation certainly represents the beating heart of GBM. Optimizing radiotherapy and its functional variables using target therapy against specific molecular actors, such as SRC, represents a promising path that needs to be smoothed out in the shortest possible time.

## Abbreviations

BPA	Boron phenylalanine
BSH	Sodium borocaptate
Cx43	Connexin43
DDR	DNA damage response
ECM	Extracellular matrix
EGF	Epidermal growth factor receptor
EMT	Epithelial–mesenchymal transition
EPO	Erythropoietin
FAK	Focal adhesion kinases
GBM	Glioblastoma
HIF-1α	Hypoxia-inducible factor-1α
IR	Ionizing radiation
LET	Linear energy transfer
MMPs	Matrix metalloproteases
OER	Oxygen enhancement ratio
ROS	Reactive oxygen species
RTKs	Receptors tyrosine kinases
SH	SRC homology domains
SRC	SRC proto-oncogene non-receptor tyrosine kinase
TIMPs	Tissue inhibitor of metalloproteases
VEGFR	Vascular endothelial growth factor receptor
